# A Hidden Transhydrogen Activity of a FMN-Bound Diaphorase under Anaerobic Conditions

**DOI:** 10.1371/journal.pone.0154865

**Published:** 2016-05-04

**Authors:** John Collins, Ting Zhang, Scott Huston, Fangfang Sun, Y.-H. Percival Zhang, Jinglin Fu

**Affiliations:** 1 Department of Chemistry, Rutgers University-Camden, Camden, New Jersey 08102, United States of America; 2 Center for Computational and Integrative Biology, Rutgers University-Camden, Camden, New Jersey 08102, United States of America; 3 Cell Free Bioinnovations Inc., Blacksburg, Virginia 24060, United States of America; 4 Department of Biological Systems Engineering, Virginia Tech, Blacksburg, Virginia 24061, United States of America; Wuhan University of Science and Technology, CHINA

## Abstract

**Background:**

Redox cofactors of NADH/NADPH participate in many cellular metabolic pathways for facilitating the electron transfer from one molecule to another in redox reactions. Transhydrogenase plays an important role in linking catabolism and anabolism, regulating the ratio of NADH/NADPH in cells. The cytoplasmic transhydrogenases could be useful to engineer synthetic biochemical pathways for the production of high-value chemicals and biofuels.

**Methodology/Principal Findings:**

A transhydrogenase activity was discovered for a FMN-bound diaphorase (DI) from *Geobacillus stearothermophilus* under anaerobic conditions. The DI-catalyzed hydride exchange were monitored and characterized between a NAD(P)H and a thio-modified NAD^+^ analogue. This new function of DI was demonstrated to transfer a hydride from NADPH to NAD^+^ that was consumed by NAD-specific lactate dehydrogenase and malic dehydrogenase.

**Conclusions/Significance:**

We discover a novel transhydrogenase activity of a FMN-DI by stabilizing the reduced state of FMNH_2_ under anaerobic conditions. FMN-DI was demonstrated to catalyze the hydride transfer between NADPH and NAD^+^. In the future, it may be possible to incorporate this FMN-DI into synthetic enzymatic pathways for balancing NADH generation and NADPH consumption for anaerobic production of biofuels and biochemicals.

## Introduction

Cellular metabolism uses many cofactors for facilitating the electron transfer from one molecule to another in redox reactions. Although chemically similar, nicotinamide adenine dinucleotide (NAD^+^) and nicotinamide adenine dinucleotide phosphate (NADP^+^) serve distinct biochemical functions in metabolism. NADH mainly participates in catabolism and provides reducing power for oxidative phosphorylation (electron-transport chains in mitochondria), generating ATP from ADP.[1] Conversely, NADPH exclusively drives the anabolic synthesis of important biomolecules, such as lipids, amino acids and sugars,[2,3] as well as the reduction of glutathione.[4] Transhydrogenase plays an important role in linking catabolism and anabolism, regulating the ratio of NADH/NADPH in cells.[5] Proton-translocating transhydrogenases are also important in bioenergetics, where the hydride transfer from a NADH to a NADP^+^ is powered by an electrochemical proton gradient in mitochondria.[3,6] Though important, many of natural transhydrogenases are membrane-bound proteins with poor solubility and low stability in aqueous solution.[5] Several efforts have been reported to express and purify soluble transhydrogenases with improved stability.[7–9] The discovery of novel cytoplasmic transhydrogenases could find utility in a number of synthetic biology applications, such as metabolic engineering and the production of high-value chemicals and biofuels.

**Fig 1:**
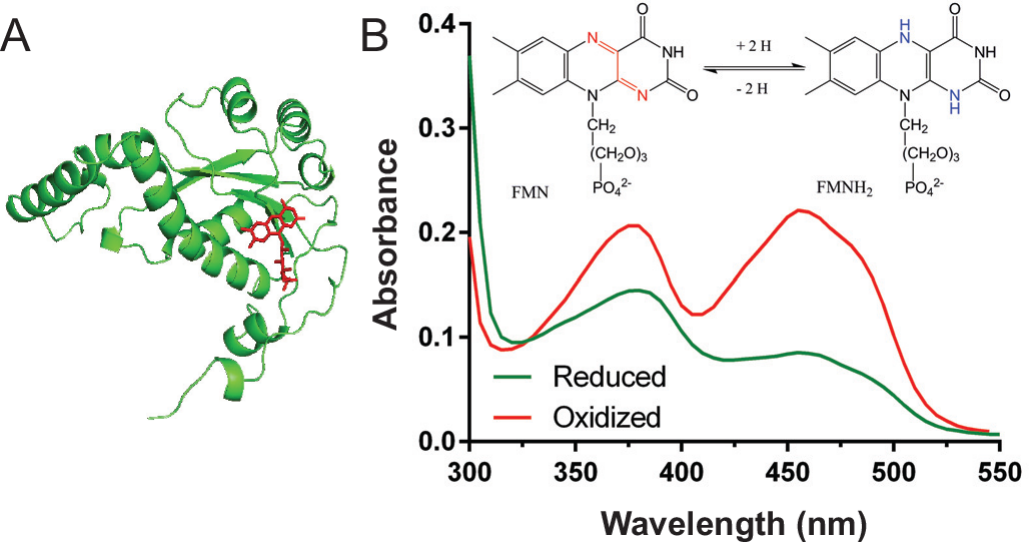
(A) An example crystal structure of a FMN-bound DI monomer (FMN is labelled in red) and (B) the decreased absorbance at 452 nm due to the reduction of FMN-DI to FMNH_2_-DI. 20 μM FMN-DI was incubated with 20 μM NADH in 1 × TBS buffer (pH 7.4).

Diaphorase (DI), a soluble NAD(P)H dehydrogenase (EC 1.6.99.1 or EC 1.6.99.3), has been found to catalyze the electron transfer from a NAD(P)H to a variety of electron acceptors, such as methylene blue,[10] resazurin,[11] vitamin K_3_,[12] azo dyes[13] and AQDS.[14] As shown in Fig 1A, a flavin mononucleoide (FMN) is bound to a protein monomer, which serves as a redox center for catalyzing the electron transfer.[15] An oxidized FMN-DI has a strong absorbance at 452 nm (Fig 1B), and can be reduced to a FMNH_2_-DI by accepting two electrons from a NAD(P)H. The reduced FMNH_2_-DI can then donate a pair of electrons to an electron acceptor. Due to its versatile functions as a NAD(P)H dehydrogenase, FMN-DI has been widely applied to the redox sensing of cofactors and enzymatic fuel cells.[12,16,17] FMN-DI also shows a NAD(P)H oxidase (EC 1.6.3.1) activity under aerobic conditions,[18–20] where a molecular oxygen is reduced to H_2_O_2_ by accepting electrons from NAD(P)H. Here we report for the first time that a FMN-DI, under anaerobic conditions (i.e., the removal of dissolved oxygen in an aqueous solution), can function as a transhydrogenase that catalyzes the exchange of a hydride between NADH and NADPH.

## Results

**Fig 2:**
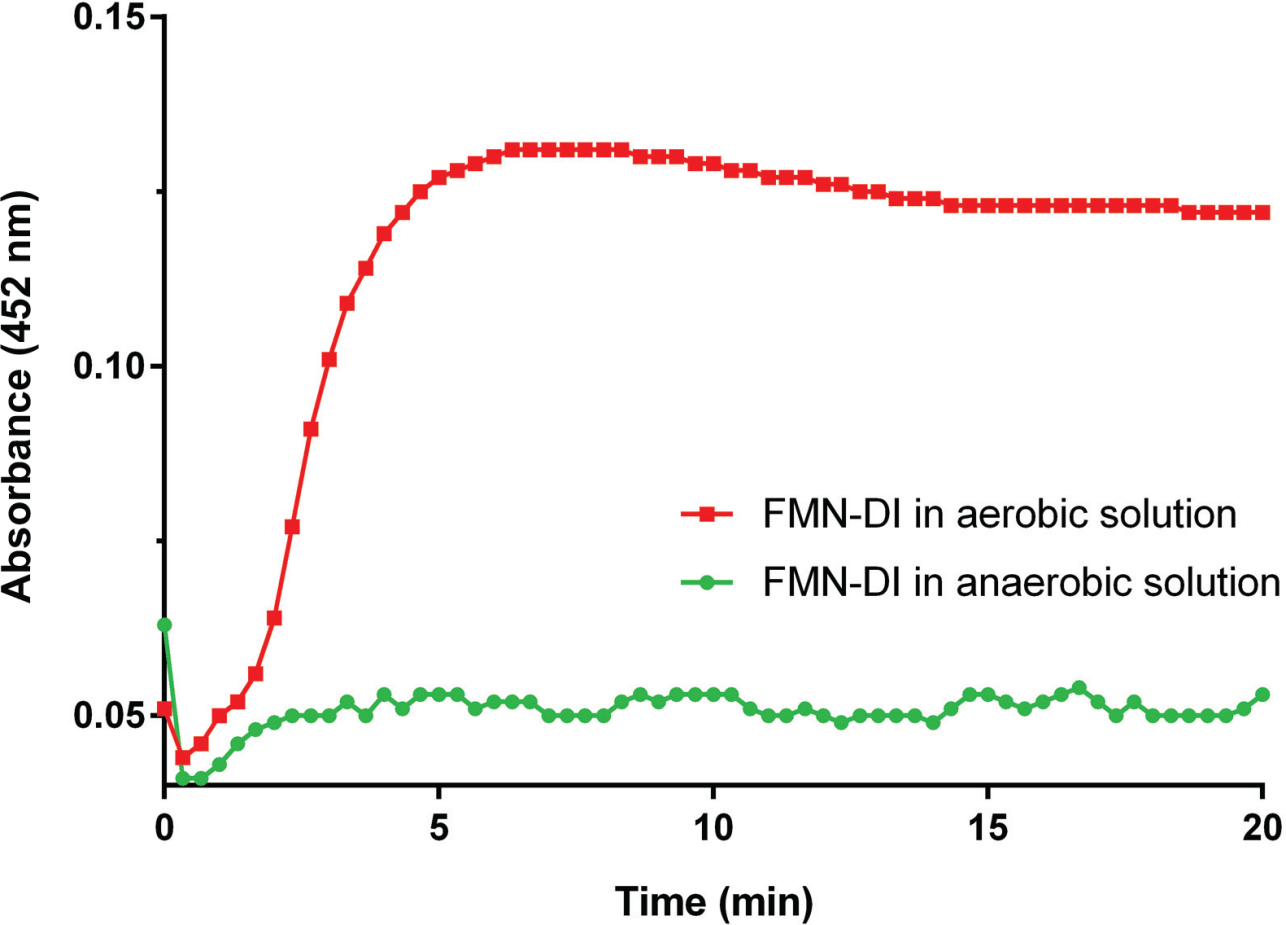
The oxidation state of FMN-DI in aerobic (red) and anaerobic (green) solution. To initiate the reduction state, 20 μM FMN-DI was first incubated with 20 μM NADH in 1 × TBS buffer (pH 7.4). The oxidation state of FMN-DI was monitored by the increased absorbance at 452 nm.

**Fig 3:**
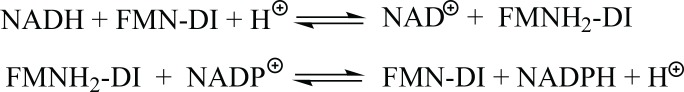
Proposed mechanism of FMN-DI catalyzing the hydride transfer between NAD(H) and NADP(H).

**Fig 4:**
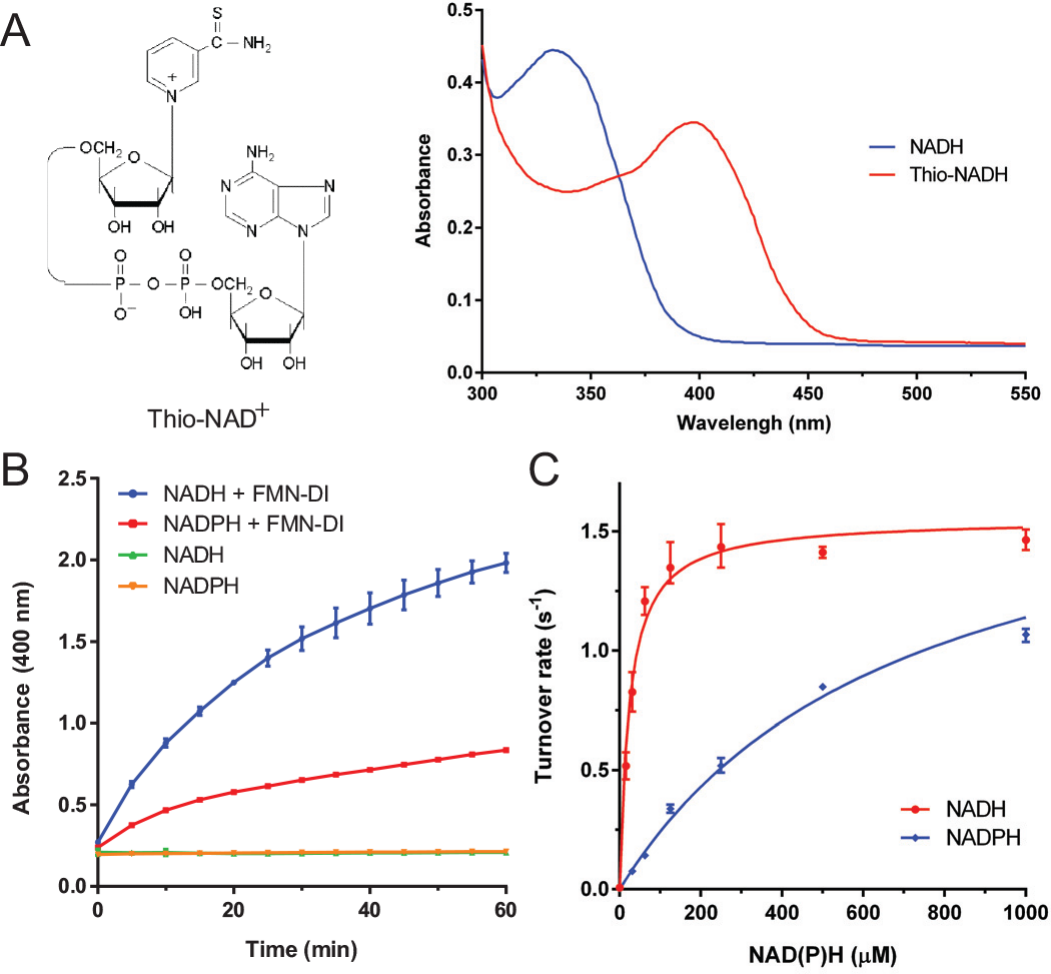
Characterization of the transhydrogenase activity of a FMN-DI. (A) A thio-NAD^+^ analogue (left) and a red-shifted maximum absorbance at ~ 400 nm for a reduced thio-NADH (right). (B) The hydride transfer from NAD(P)H to thio-NAD^+^ with the increased absorbance at 400 nm. Condition: 500 nM FMN-DI was incubated with 500 μM thio-NAD^+^ and 500 μM NAD(P)H in 1 × TBS buffer (pH 7.4) at room temperature. (C) The Michaelis-Menten fitting of NAD(P)H concentrations for the DI-catalyzed transhydrogen reaction. Error bars were generated as the range of at least three replicates.

The recombinant FMN-DI from *Geoba*cillus *stearothermophilus* was expressed in *E*. *coli* and purified as previously reported.[16] As shown in Fig 2, a 20 μM solution of FMN-DI was first reduced to FMNH_2_-DI by adding 20 μM NADH. In regular aqueous solution containing dissolved oxygen, FMNH_2_-DI was quickly oxidized back to FMN-DI with the simultaneous reduction of oxygen to H_2_O_2_, [15,17] thus resulting in the increased absorbance at 452 nm. To stabilize the reduced state of FMNH_2_-DI, dissolved oxygen was removed by purging the aqueous buffer solution with pure argon gas (20 psi, 30 min).[21,22] Under this anaerobic condition, FMNH_2_-DI maintained a stable reduction state with a low absorbance at 452 nm. We hypothesized that FMN-DI, with the stabilized reduction state, might perform as a transhydrogenase which catalyzes the hydride exchange between a NADPH and a NAD^+^ (or vice versa). As shown in Fig 3, FMN-DI serves as a redox center to catalyze the reversible transfer of hydrides. To test this, analogues of thio-NAD^+^ and thio-NADP^+^ were used to characterize the transhydrogen reaction.[7,23,24] As shown in Fig 4A, a thio-NAD^+^ analogue resembles the structure of a NAD^+^, except for a thio-ester substitution at the nicotinamide group, which exhibits a red-shifted absorbance for reduced thio-NADH at ~ 400 nm. This shifted absorbance of thio-NADH is easily distinguishable from the 340 nm absorbance of NAD(P)H(Fig 4B). We first compared the substrate activity of the two analogues of thio-NAD^+^ and thio-NADP^+^ for FMN-DI. It was found that thio-NADP^+^ was much less active than thio-NAD^+^ for FMN-DI (S1 Fig). Further kinetics study showed that FMN-DI exhibited a much smaller *k*_*cat*_ for thio-NADP^+^ than that for thio-NAD^+^ (S2 Fig, S3 Fig, S4 Fig and S5 Fig). Thus only thio-NAD^+^ was used as a reporter for characterizing the transhydrogen reaction. As shown in Fig 4B, the increased absorbance at 400 nm was observed for the DI-catalyzed transhydrogen reaction between NADH and thio-NAD^+^, as well as between NADPH and thio-NAD^+^. As a negative control, the incubation of thio-NAD^+^ with NADH or NADPH did not result in a significant increase of absorbance at 400 nm. Another control experiment also showed that the addition of free FMN molecules could not enhance the rate of the transhydrogen reaction (S6 Fig and S7 Fig). All of these results suggested that the DI-bound FMN cofactor was responsible for catalyzing the transhydrogen reaction, not the unbound and freely diffused FMN. FMN-DI was further found to discriminate between NADH and NADPH, which catalyzed the reaction between NADH and thio-NAD^+^ 3-fold faster than the reaction between NADPH and thio-NAD^+^. Detailed kinetic analysis showed that FMN-DI exhibited similar turnover numbers between NADH (a *k*_*cat*_ value of ~ 1.64 ± 0.05 s^-1^) and NADPH (a *k*_*cat*_ value of ~ 1.93 ± 0.06 s^-1^). However, the apparent *K*_*m*_ value of NADH (~ 24 ± 3 μM) was much smaller than that of NADPH (~ 699 ± 49 μM) (Fig 3C). This suggested that NADPH was bound to the enzyme less efficiently than NADH. Thus FMN-DI might favor the hydride transfer from a high concentration of NADPH to a low concentration of NAD^+^. Detailed activity curves are shown in S8 Fig, S9 Fig and S10 Fig.

**Fig 5:**
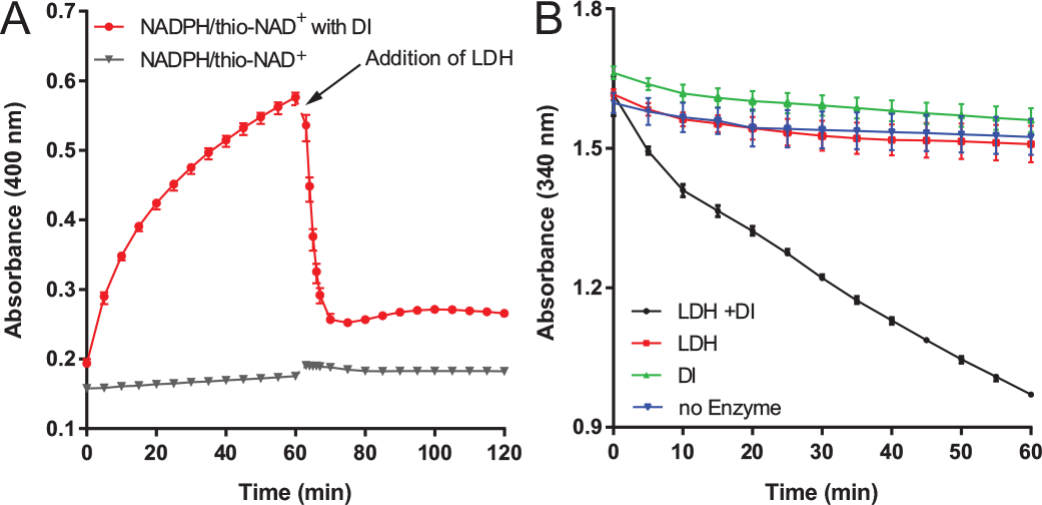
(A) Real-time monitoring of the hydride exchange from NADPH to thio-NAD^+^ at 400 nm, and the consumption of thio-NADH with the addition of LDH. Condition: 1 mM NADPH and 1 mM thio-NAD^+^ were first incubated with 500 nM FMN-DI in 1 × TBS buffer (pH 7.4) at room temperature. Then 10 nM LDH and 1 mM pyruvate were added to oxidize thio-NADH. (B) The LDH-catalyzed oxidation was activated by the addition of a FMN-DI to convert NADPH to NADH (black) and controls of no addition of DI (red), no addition of LDH (red) and no addition of enzymes (blue). Condition: 1 mM NADPH and 1 mM NAD^+^ were first incubated with or without 1 μM FMN-DI for one hour in 1 × TBS buffer (pH 7.4). Then 1 nM LDH and 1 mM pyruvate were added to evaluate the oxidation of the mixture of NADPH and NAD^+^. Anaerobic solution was used for the assay. Error bars were generated as the range of at least three replicates.

Using this newly discovered transhydrogenase activity, FMN-DI was demonstrated to allow a NADH-specific lactate dehydrogenase to utilize NADPH as an electron donor, where NADPH was converted to NADH by the DI-catalyzed transhydrogen reaction. We chose a special lactate dehydrogenase (LDH) that reacted with NADH 100-fold faster than NADPH for converting pyruvate to lactate (S11 Fig). As shown in Fig 5A, we first used a mixture of NADPH and thio-NAD^+^, where the DI-catalyzed the hydride exchange from NADPH to thio-NAD^+^, generating thio-NADH accompanied with an increased absorbance at 400 nm. The sequential addition of LDH induced a quick decrease in the absorbance at 400 nm, indicating the consumption of thio-NADH by LDH. Next, we tested FMN-DI for catalyzing the direct hydride transfer between NADPH and NAD^+^. As shown in Fig 5B, LDH cannot efficiently use NADPH as an electron donor without the addition of FMN-DI, resulting in a very slow decrease in the absorbance at 340 nm (shown in red). Conversely, the addition of FMN-DI into the reaction mixture catalyzed the hydride transfer from NADPH to NAD^+^ with the production of more NADH. Then, the produced NADH was quickly consumed by the LDH with a faster decreased absorbance at 340 nm (shown in black). As another control experiment, FMN-DI was incubated with the mixture of NADPH and NAD^+^ without the addition of LDH to consume the produced NADH (shown in green). The absorbance at 340 nm varied slightly over time which was similar to that of the no-enzyme control (shown in blue) because both reduced NADH and NADPH had similar absorbance at 340 nm. Similarly, FMN-DI was also demonstrated to activate a NADH-specific malic dehydrogenase to utilize NADPH for reducing oxaloacetate (S12 Fig, S13 Fig and S14 Fig). The above results demonstrated that FMN-DI catalyzed the hydride exchange between NADPH and NAD^+^ under anaerobic conditions. Most natural transhydrogenases in living cells under reduced environments favor the transhydrogenation from NADPH to NADH, mainly due to the fact that the physiological ratio of NADPH/NADP^+^ (~ 60) is much higher than the ratio of NADH/NAD^+^ (~ 0.03),[5,7] and the cellular concentration of NADPH (~ 120 μM) is also higher than that of NADH (~ 80 μM).[25] This study implied that DI could have a new function as transhydrogenase for some organisms, especially for anaerobic species.

## Discussion

In summary, we discover a novel transhydrogenase activity of FMN-DI by stabilizing the reduced state of FMNH_2_ under anaerobic conditions. FMN-DI was demonstrated to catalyze the hydride transfer between NADPH and NAD^+^. In the future, it may be possible to incorporate this FMN-DI into synthetic enzymatic pathways for balancing NADH generation and NADPH consumption for anaerobic production of biofuels and biochemicals.

## Materials and Methods

### Chemicals

Solid Tris base, 10x Tris buffered saline (TBS) were purchased from Fisher Scientific (Waltham, MA). Reduced β-Nicotinamide adenine dinucleotide (NADH), reduced β-Nicotinamide adenine dinucleotide phosphate (NADPH), β-Nicotinamide adenine dinucleotide (NAD^+^), Lactate Dehydrogenase from rabbit muscle (LDH), and sodium pyruvate were all purchased from Sigma Aldrich (St. Louis, MO). Thio-NAD^+^, thio-NADH, and thio-NADP+ were all purchased from Oriental Yeast Company (Tokyo, Japan). Argon gas (Part #: AR 4.8OF-Q) was purchased from Praxair (Philadelphia, PA). Captair pyramid 2200 multi-function disposable glove box (size = XL) was purchased from Erlab (Rowley, MA).

### Expression of Diaphorase

The procedures for enzyme expression and purification are similar as described previously.[16] Briefly, the 636-bp DNA fragment encoding diaphorase (DI, GenBank accession number JQ040550) was expressed in *E*.*coli*. The DNA was amplified by PCR using the genomic DNA of G. stearothermophilus 10 as the template and two primers (forward primer: 50 -ACT TTA AGA AGG AGA TAT ACA TAT GAC GAA AGT ATT GTA CAT CAC CGC CC-30; reverse primer: 50 -AGT GGT GGT GGT GGT GGT GCT CGA GAA ACG TGT GCG CCA AGT CTT TCG CC-30). The recombinant GsDI (briefly called DI) was expressed in the plasmid pET20b-Gsdi, which was obtained using Simple Cloning.[26] The recombinant plasmids were transformed into *E*. *coli* BL21 Star (DE3). The expression of the recombinant protein was induced by adding isopropyl b-D-1- thiogalactopyranoside (IPTG) (0.1 mM final concentration). The cultures were incubated at 18°C for 16 h. The cells were harvested by centrifugation at 4°C. The collected cells were disrupted by sonication, and the soluble target protein in the supernatant of the crude extract was purified using a Bio-Rad Profinity IMAC Ni-Charged Resin (Hercules, CA).[27] The purified FMN-DI concentration was determined by the molar extinction coefficient of bound FMN, which was ~ 12500 cm^-1^ M^-1^ at 455 nm. [28,29]

### Preparation of anaerobic environment for enzyme assay

All sample preparations were performed in an argon-protected pyramid glove box. Solutions of enzymes, substrates and buffers, pipettes and 96-well plates were placed inside a pyramid glove box. The pyramid glove box was first fully filled with argon gas from the bottom, and then all the gas was purged out. The argon purging was repeated twice to ensure that air inside the box was mostly replaced with argon. The glove box was filled with argon gas and was well sealed. The buffer solutions were bubbled for ~ 20 min at ~ 20 psi with argon to remove dissolved oxygen immediately prior to the assay measurement. The 96-well plate was covered by an optically transparent lid, and sealed with vacuum grease (Dow Corning, MI) on the sides. After pipetting the sample solution, the sealed 96-well plate was transferred to a Cytation 3 Cell Imaging Multi-Mode Reader (Biotek, VT) for enzymatic assay.

### Enzyme assay

All enzyme assays were carried out using a Cytation 3 Cell Imaging Multi-Mode Reader (Biotek, VT). For reactions involving NAD(P)H, the absorbance at 340 nm was monitored in real time. Thio-modified analogues (thio-NAD^+^/thio-NADP^+^) were monitored at 400 nm. At least three replicates were tested in parallel. The rate of the enzyme-catalyzed reaction was determined by fitting the initial velocity of curves, and the Michaelis-Menten constants were determined by fitting enzyme activities as a function of substrate concentrations, using the equation: *Y = V*_max_*X/(K_*m*_*+X)*, where *Y* is rate of the reaction, and *X* is the substrate concentration. All fittings were preformed using GraphPad Prism 6. The fitted *V*_*max*_ was converted to turnovers (s^-1^) using the calibration curves as shown in S9 Fig.

## Supporting Information

S1 Fig Substrate activity of thio-NAD^+^ and thio-NADP^+^ for a FMN-DI. (PDF)

S2 Fig Raw activity curves of titrating the concentration of thio-NAD^+^. (PDF)

S3 Fig The Michaelis-Menten fitting of thio-NAD^+^ concentrations for the DI-catalyzed transhydrogen reaction. (PDF)

S4 Fig Raw activity curves of titrating the concentration of thio-NADP^+^. (PDF)

S5 Fig The Michaelis-Menten fitting of thio-NADP^+^ concentrations for the DI-catalyzed transhydrogen reaction. (PDF)

S6 Fig The addition of free FMN molecules does not significantly catalyze the transhydrogen reaction between a NADH and a thio-NAD^+^. (PDF)

S7 Fig The slopes of reaction curves containing 0, 1, 10 and 100 μM FMN were similar. (PDF)

S8 Fig Raw curves used for Michaelis-Menten kinetic fitting of the *K*_*m*_ and *k*_*cat*_ values for NADH. (PDF)

S9 Fig Raw curves used for Michaelis-Menten kinetic fitting of the *K*_*m*_ *and k*_*cat*_ values for NADPH. (PDF)

S10 Fig Standard calibration curve of OD400 values *vs* thio-NADH concentration. (PDF)

S11 Fig Activity of LDH toward NADH and NADPH. (PDF)

S12 Fig Raw activity curves for comparing the activity of MDH for NADH and NADPH. (PDF)

S13 Fig Real-time monitoring of the hydride exchange from NADPH to thio-NAD^+^. (PDF)

S14 Fig The MDH-catalyzed oxidation was activated by the addition of a FMN-DI to convert NADPH to NADH. (PDF)
