# Supplementary material for: A Hidden Transhydrogen Activity of a FMN-Bound Diaphorase under Anaerobic Conditions
Source: PLoS One. 2016 May 4;11(5):e0154865. doi: 10.1371/journal.pone.0154865 (PMC4856307; doi:10.1371/journal.pone.0154865)
Supplement: S1 Fig — (PDF) [file pone.0154865.s001.pdf]

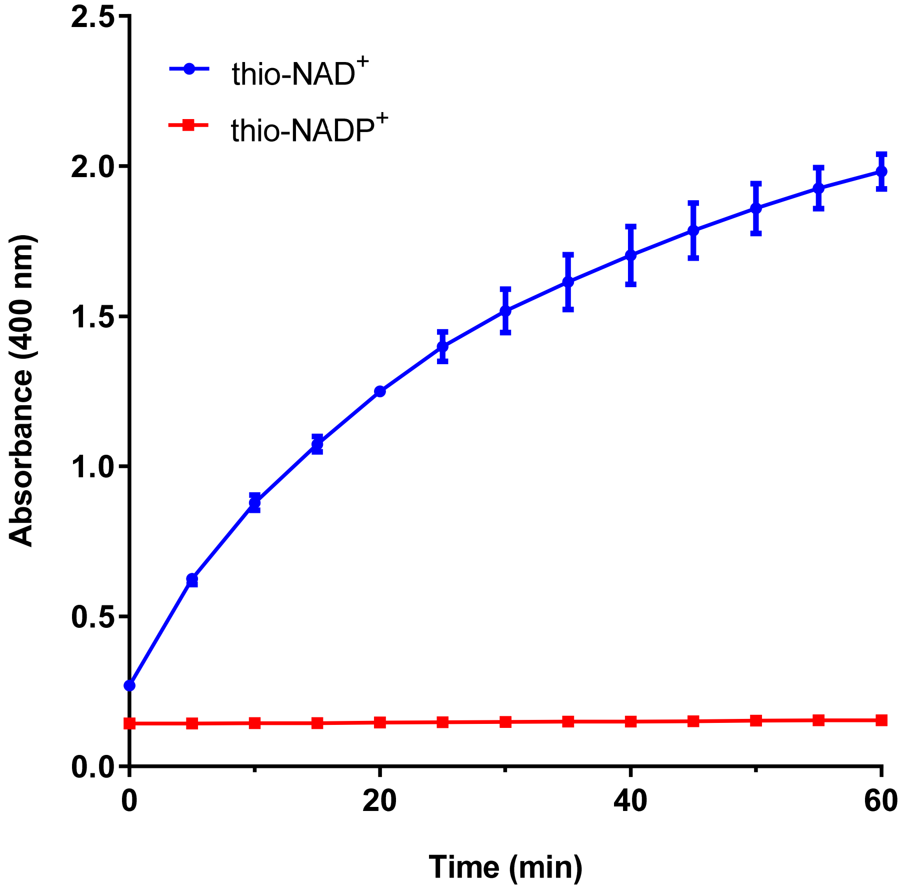

S1 Fig. Substrate activity of thio-NAD$^+$ and thio-NADP$^+$ for a FMN-DI. A hydride transfer between NADH and thio-NAD(P)$^+$ was monitored by an increased absorbance at 400 nm. Conditions: 500 nM DI was added into a solution containing 500 μM NADH and either 500 μM thio-NAD$^+$ (blue) or thio-NADP$^+$ (red) in 1 × TBS (pH 7.4) at room temperature. The thio-NADP$^+$ was found to have almost no activity with a FMN-DI. Error bars were generated as the range of at least three replicates.
