# Supplementary material for: A Hidden Transhydrogen Activity of a FMN-Bound Diaphorase under Anaerobic Conditions
Source: PLoS One. 2016 May 4;11(5):e0154865. doi: 10.1371/journal.pone.0154865 (PMC4856307; doi:10.1371/journal.pone.0154865)
Supplement: S2 Fig — (PDF) [file pone.0154865.s002.pdf]

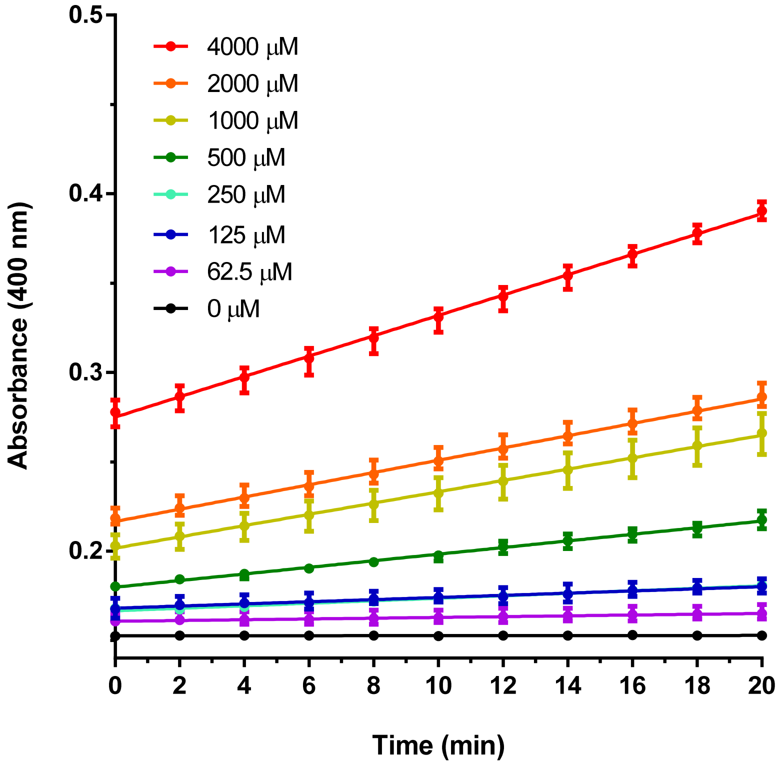

S2 Fig. Raw activity curves of titrating the concentration of thio-NAD$^+$. The hydride transfer between NADH and thio-NAD$^+$ was monitored by the increased absorbance at 400 nm. Conditions: 2 mM NADH and 25 nM DI were incubated with a set of thio-NAD$^+$ concentrations varied from 62.5 μM to 4000 μM, in pH 7.4, 1 × TBS buffer at room temperature. Error bars were generated as the range of at least three replicates.
