# Supplementary material for: A Hidden Transhydrogen Activity of a FMN-Bound Diaphorase under Anaerobic Conditions
Source: PLoS One. 2016 May 4;11(5):e0154865. doi: 10.1371/journal.pone.0154865 (PMC4856307; doi:10.1371/journal.pone.0154865)
Supplement: S3 Fig — (PDF) [file pone.0154865.s003.pdf]

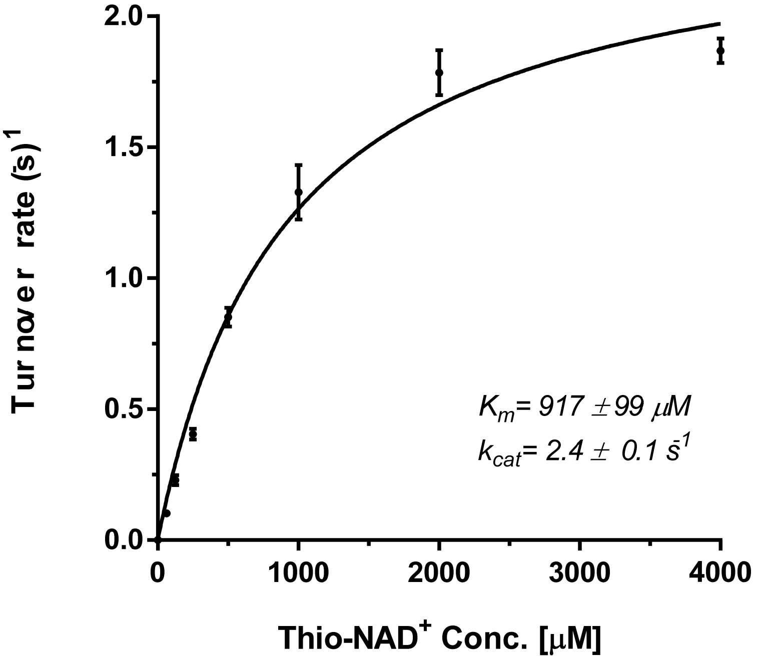

S3 Fig. The Michaelis-Menten fitting of thio-NAD$^+$ concentrations for the DI-catalyzed transhydrogen reaction. Error bars were generated as the range of at least three replicates.
