# Supplementary material for: A Hidden Transhydrogen Activity of a FMN-Bound Diaphorase under Anaerobic Conditions
Source: PLoS One. 2016 May 4;11(5):e0154865. doi: 10.1371/journal.pone.0154865 (PMC4856307; doi:10.1371/journal.pone.0154865)
Supplement: S4 Fig — (PDF) [file pone.0154865.s004.pdf]

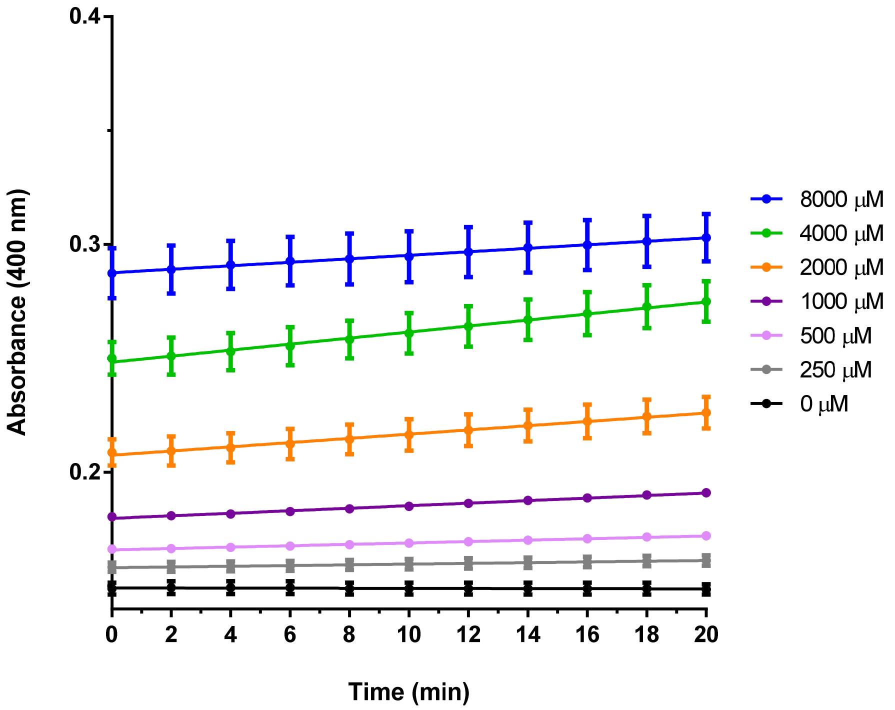

S4 Fig. Raw activity curves of titrating the concentration of thio-NADP$^+$. The hydride transfer between NADH and thio-NADP$^+$ was monitored by the increased absorbance at 400 nm. Conditions: 2 mM NADH and 100 nM DI were incubated with a set of thio-NADP$^+$ concentrations varied from 250 μM to 8000 μM, in pH 7.4, 1 × TBS buffer at room temperature. Error bars were generated as the range of at least three replicates. Thio-NADP$^+$ is poorly reacted with a FMN-DI, with clearly observed activities for the concentrations > 1000 μM. Substrate inhibition also happened for thio-NADP$^+$ at 8000 μM or above.
