# Supplementary material for: A Hidden Transhydrogen Activity of a FMN-Bound Diaphorase under Anaerobic Conditions
Source: PLoS One. 2016 May 4;11(5):e0154865. doi: 10.1371/journal.pone.0154865 (PMC4856307; doi:10.1371/journal.pone.0154865)
Supplement: S5 Fig — (PDF) [file pone.0154865.s005.pdf]

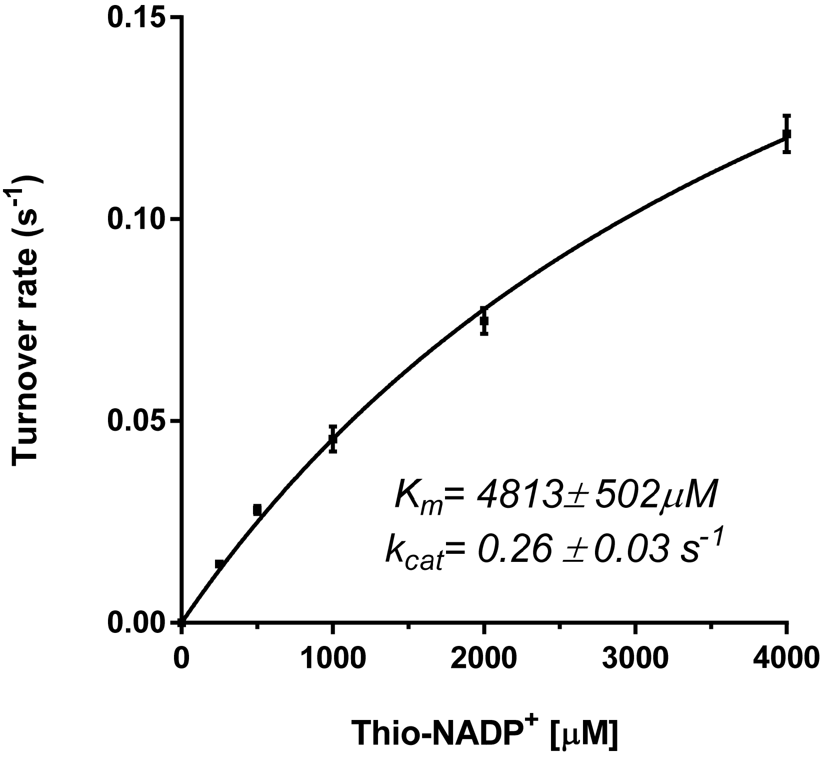

S5 Fig. The Michaelis-Menten fitting of thio-$NADP^+$ concentrations for the DI-catalyzed transhydrogen reaction. Error bars were generated as the range of at least three replicates.

As shown in S4 Fig and S5 Fig, thio-$NADP^+$ is poorly interacted with a FMN-DI, with a larger K_m (~ 4813 µM, the K_m estimation is affected by the substrate inhibition at the high concentration) and a very smaller k_{cat} (~ 0.26 s^{-1}), as compared to the values of thio-NAD^+ (K_m ~ 917 µM and k_{cat} ~ 2.4 s^{-1}) shown in S2 Fig and S3 Fig.
