# Supplementary material for: A Hidden Transhydrogen Activity of a FMN-Bound Diaphorase under Anaerobic Conditions
Source: PLoS One. 2016 May 4;11(5):e0154865. doi: 10.1371/journal.pone.0154865 (PMC4856307; doi:10.1371/journal.pone.0154865)
Supplement: S6 Fig — (PDF) [file pone.0154865.s006.pdf]

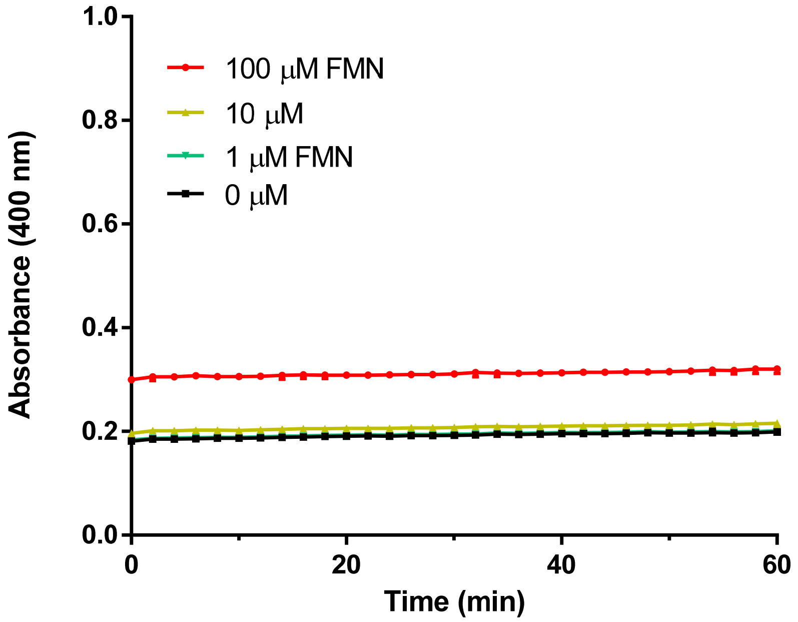


**S6 Fig.** The addition of free FMN molecules does not significantly catalyze the transhydrogen reaction between a NADH and a thio-NAD$^+$. Condition: FMN from 1 μM to 100 μM were added into a solution containing 1 mM NADH and 1 mM thio-NAD$^+$ in pH 7.4, 1 × TBS at room temperature. The slopes of all curves were similar (as shown in S7 Fig). Error bars were generated as the range of at least three replicates.
