# Supplementary material for: A Hidden Transhydrogen Activity of a FMN-Bound Diaphorase under Anaerobic Conditions
Source: PLoS One. 2016 May 4;11(5):e0154865. doi: 10.1371/journal.pone.0154865 (PMC4856307; doi:10.1371/journal.pone.0154865)
Supplement: S7 Fig — (PDF) [file pone.0154865.s007.pdf]

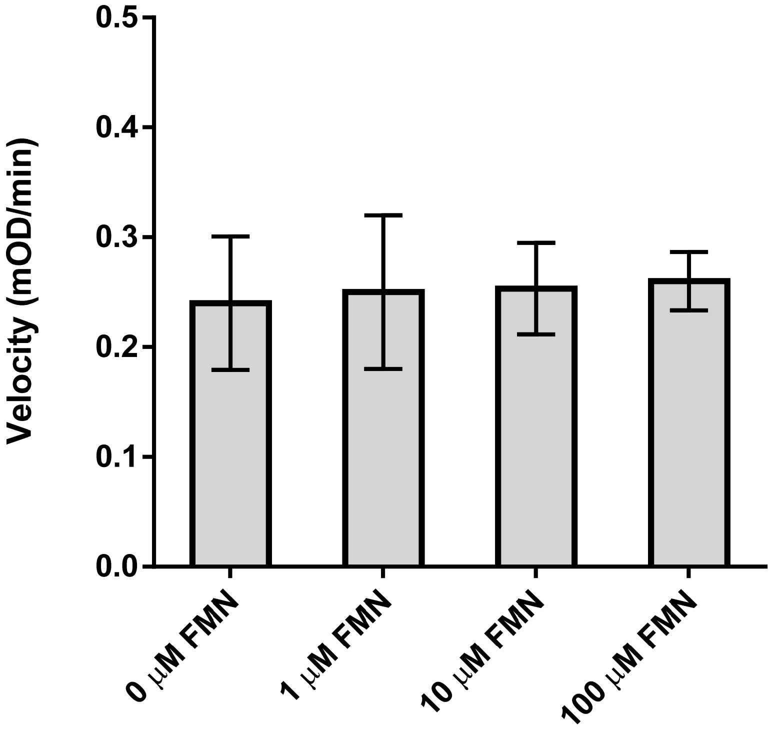

S7 Fig. The slopes of reaction curves (as shown in S6 Fig) containing 0, 1, 10 and 100 μM FMN were similar. This indicates that the addition of free FMN molecules does not significantly catalyze the transhydrogen reaction between a NADH and a thio-NAD^+. Error bars were generated as the range of at least three replicates.
