# Supplementary material for: A Hidden Transhydrogen Activity of a FMN-Bound Diaphorase under Anaerobic Conditions
Source: PLoS One. 2016 May 4;11(5):e0154865. doi: 10.1371/journal.pone.0154865 (PMC4856307; doi:10.1371/journal.pone.0154865)
Supplement: S8 Fig — (PDF) [file pone.0154865.s008.pdf]

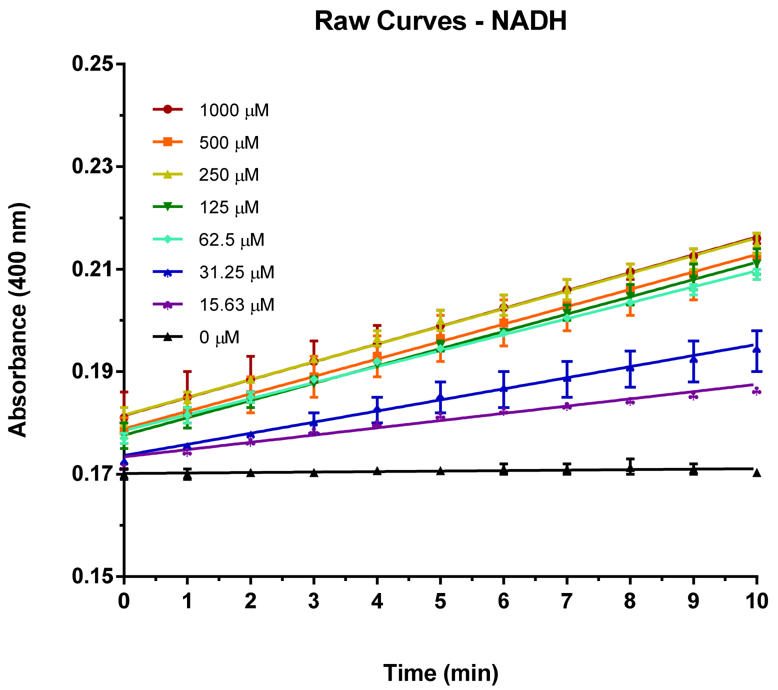

S8 Fig. Raw curves used for Michaelis-Menten kinetic fitting of the K_m and k_{cat} values for NADH. The hydride transfer between NADH and thio-NAD^+ was monitored by the increased absorbance at 400 nm. Initial velocities were determined by fitting the linear range of the beginning 5-10 minutes of the reaction. Conditions: 2 mM thio-NAD^+ and 25 nM DI were incubated with a set of NADH concentrations varied from 15 μM to 1000 μM, in pH 7.4, 1 × TBS buffer at room temperature. Error bars were generated as the range of at least three replicates.
