# Supplementary material for: A Hidden Transhydrogen Activity of a FMN-Bound Diaphorase under Anaerobic Conditions
Source: PLoS One. 2016 May 4;11(5):e0154865. doi: 10.1371/journal.pone.0154865 (PMC4856307; doi:10.1371/journal.pone.0154865)
Supplement: S9 Fig — (PDF) [file pone.0154865.s009.pdf]

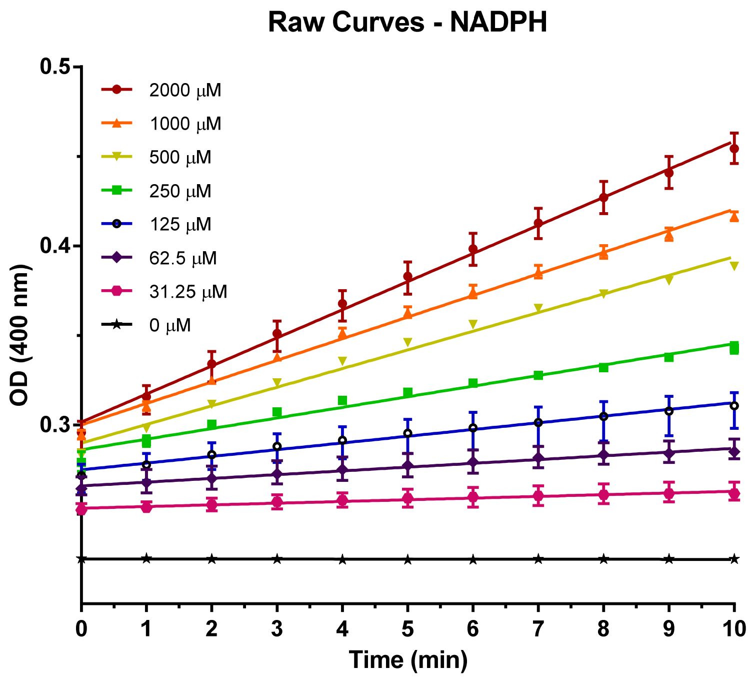

S9 Fig. Raw curves used for Michaelis-Menten kinetic fitting of the K_m and k_{cat} values for NADPH. The hydride transfer between NADPH and thio-NAD^+ was monitored by the increased absorbance at 400 nm. Initial velocities were determined by fitting the linear range of the beginning 5-10 minutes of the reaction. Conditions: 2 mM thio-NAD^+ and 100 nM DI were incubated with a set of NADPH concentrations varied from 31 μM to 2000 μM, in pH 7.4, 1 × TBS buffer at room temperature. Error bars were generated as the range of at least three replicates.
