# Supplementary material for: A Hidden Transhydrogen Activity of a FMN-Bound Diaphorase under Anaerobic Conditions
Source: PLoS One. 2016 May 4;11(5):e0154865. doi: 10.1371/journal.pone.0154865 (PMC4856307; doi:10.1371/journal.pone.0154865)
Supplement: S10 Fig — (PDF) [file pone.0154865.s010.pdf]

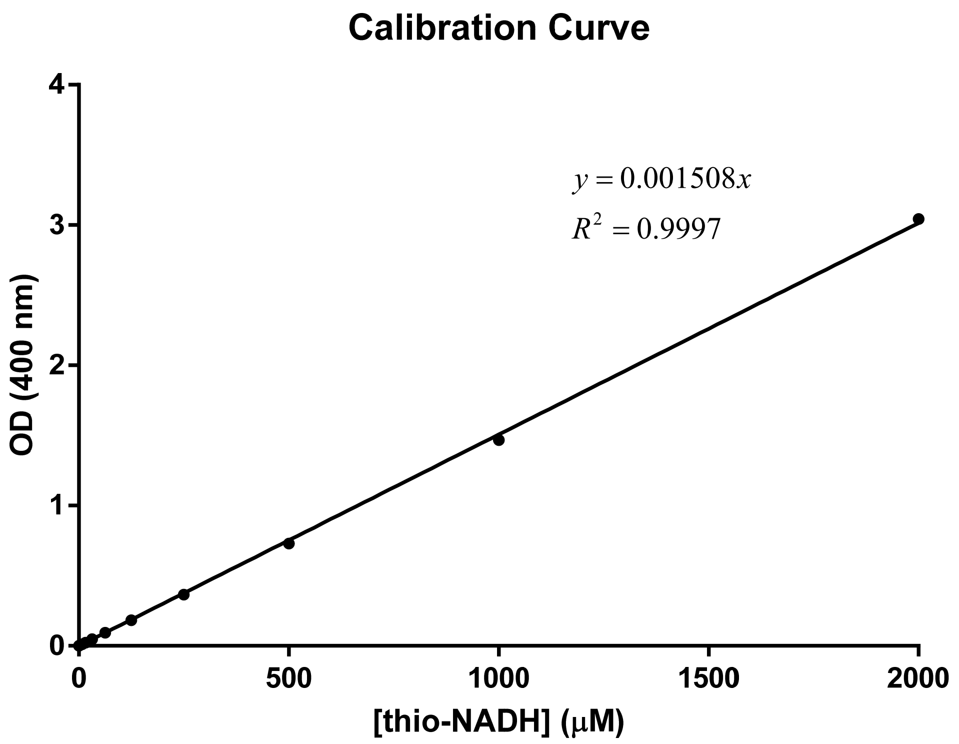

S10 Fig. Standard calibration curve of OD400 values *vs* thio-NADH concentration. The calibration curve was measured in a 96-well plate with a lid that was similar as that employed in the enzyme assay. Error bars were generated as the range of at least nine replicates.
