# Supplementary material for: A Hidden Transhydrogen Activity of a FMN-Bound Diaphorase under Anaerobic Conditions
Source: PLoS One. 2016 May 4;11(5):e0154865. doi: 10.1371/journal.pone.0154865 (PMC4856307; doi:10.1371/journal.pone.0154865)
Supplement: S11 Fig — (PDF) [file pone.0154865.s011.pdf]

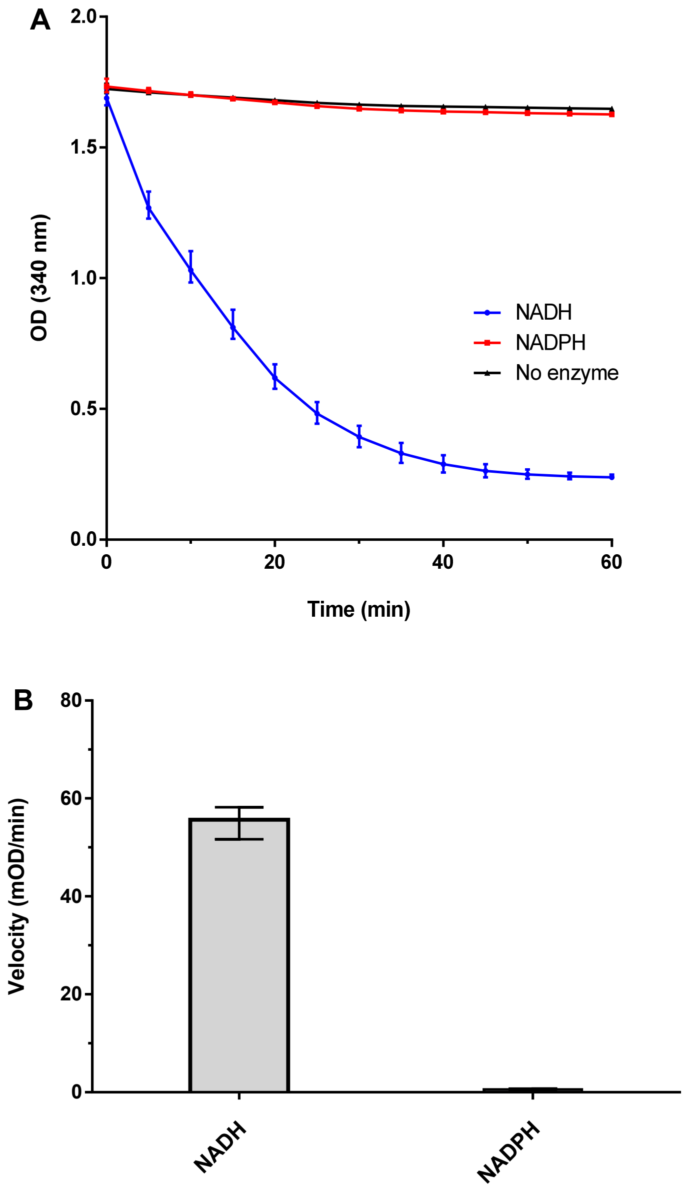

S11 Fig. Activity of LDH toward NADH and NADPH. (A) Raw activity curves for comparing the activity of LDH for NADH and NADPH. (B) The LDH-catalyzed oxidation velocity of NADH and NADPH: 1 nM LDH was added to the substrate solution of 1 mM NAD(P)H and 1 mM Pyruvate at room temperature in pH 7.4, 1×TBS buffer. Error bars were generated as the range of at least three replicates.
