# Supplementary material for: A Hidden Transhydrogen Activity of a FMN-Bound Diaphorase under Anaerobic Conditions
Source: PLoS One. 2016 May 4;11(5):e0154865. doi: 10.1371/journal.pone.0154865 (PMC4856307; doi:10.1371/journal.pone.0154865)
Supplement: S12 Fig — (PDF) [file pone.0154865.s012.pdf]

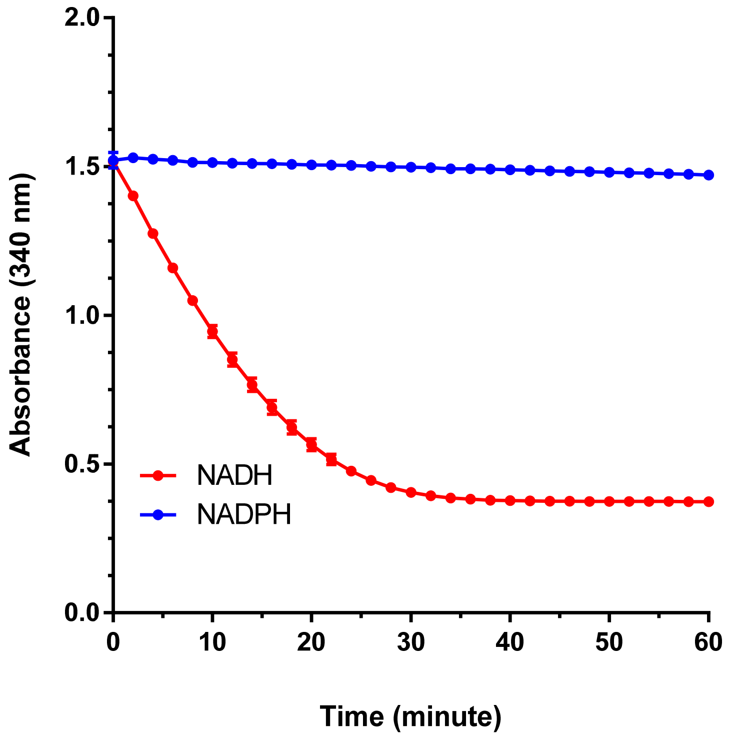

S12 Fig. Raw activity curves for comparing the activity of MDH for NADH and NADPH. Running conditions: 20 nM MDH was added to the substrate solution of 1 mM NAD(P)H and 1 mM oxaloacetate at room temperature in pH 7.4, 1×TBS buffer. Error bars were generated as the range of at least three replicates.
