# Supplementary material for: A Hidden Transhydrogen Activity of a FMN-Bound Diaphorase under Anaerobic Conditions
Source: PLoS One. 2016 May 4;11(5):e0154865. doi: 10.1371/journal.pone.0154865 (PMC4856307; doi:10.1371/journal.pone.0154865)
Supplement: S13 Fig — (PDF) [file pone.0154865.s013.pdf]

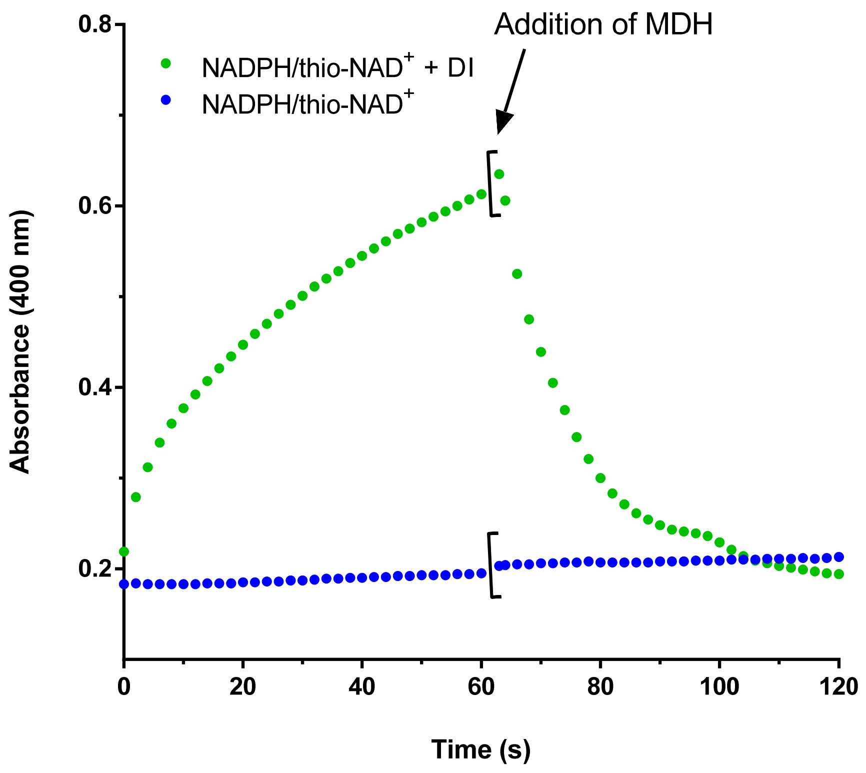

S13 Fig. Real-time monitoring of the hydride exchange from NADPH to thio-NAD$^+$ at 400 nm, and the consumption of thio-NADH with the addition of MDH. Condition: 1 mM NADPH and 1 mM thio-NAD$^+$ were first incubated with 500 nM FMN-DI in 1 × TBS buffer (pH 7.4) at room temperature. Then 100 nM MDH and 1 mM oxaloacetate were added to oxidize thio-NADH.
