# Supplementary material for: A Hidden Transhydrogen Activity of a FMN-Bound Diaphorase under Anaerobic Conditions
Source: PLoS One. 2016 May 4;11(5):e0154865. doi: 10.1371/journal.pone.0154865 (PMC4856307; doi:10.1371/journal.pone.0154865)
Supplement: S14 Fig — (PDF) [file pone.0154865.s014.pdf]

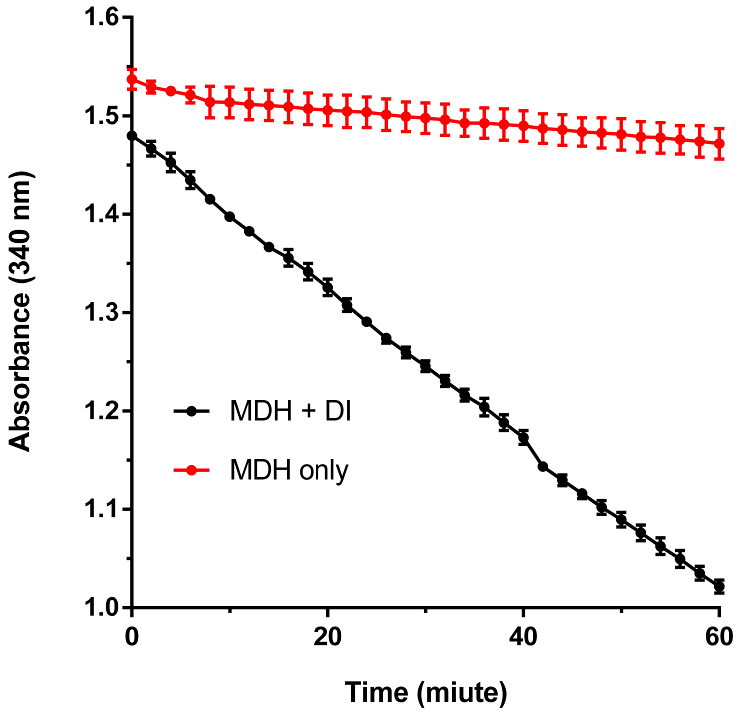

S14 Fig. The MDH-catalyzed oxidation was activated by the addition of a FMN-DI to convert NADPH to NADH (black) and controls of no addition of DI (red). Condition: 1 mM NADPH and 1 mM NAD^+ were first incubated with or without 500 nM FMN-DI for one hour in 1 × TBS buffer (pH 7.4). Then 100 nM MDH and 1 mM oxaloacetate were added to evaluate the oxidation of the mixture of NADPH and NAD^+. Anaerobic solution was used for the assay. Error bars were generated as the range of at least three replicates.
